# Myofascial Injection Using Fascial Layer-Specific Hydromanipulation Technique (FLuSH) and the Delineation of Multifactorial Myofascial Pain

**DOI:** 10.3390/medicina56120717

**Published:** 2020-12-20

**Authors:** Tina Wang, Roya Vahdatinia, Sarah Humbert, Antonio Stecco

**Affiliations:** 1 School of Medicine, Loma Linda University, Loma Linda, CA 92350, USA; rvahdatinia@atsu.edu (R.V.); Sarah.Humbert@va.gov (S.H.); 2 Department of Rehabilitation Medicine, Grossman School of Medicine, New York University, New York, NY 10016, USA; antonio.stecco@gmail.com

**Keywords:** injection, myofascial pain, ultrasound, fascia, myofascial unit

## Abstract

*Background and objectives*: The aims of this study were to delineate the contribution of specific fascial layers of the myofascial unit to myofascial pain and introduce the use of ultrasound-guided fascial layer-specific hydromanipulation (FLuSH) as a novel technique in the treatment of myofascial pain. *Materials and Methods*: The clinical data of 20 consecutive adult patients who underwent myofascial injections using FLuSH technique for the treatment of myofascial pain were reviewed. The FLuSH technique involved measuring the pain pressure threshold using an analog algometer initially and after each ultrasound guided injection of normal saline into the specific layers of the myofascial unit (superficial fascia, deep fascia, or muscle) in myofascial points corresponding with Centers of Coordination/Fusion (Fascial Manipulation^®^). The outcome measured was the change in pain pressure threshold after injection of each specific fascial layer. *Results*: Deep fascia was involved in 73%, superficial fascia in 55%, and muscle in 43% of points. A non-response to treatment of all three layers occurred in 10% of all injected points. The most common combinations of fascial layer involvement were deep fascia alone in 23%, deep fascia and superficial fascia in 22%, and deep fascia and muscle in 18% of injected points. Each individual had on average of 3.0 ± 1.2 different combinations of fascial layers contributing to myofascial pain. *Conclusions*: The data support the hypothesis that multiple fascial layers are responsible for myofascial pain. In particular, for a given patient, pain may develop from discrete combinations of fascial layers unique to each myofascial point. Non-response to treatment of the myofascial unit may represent a centralized pain process. Adequate treatment of myofascial pain may require treatment of each point as a distinct pathologic entity rather than uniformly in a given patient or across patients.

## 1. Introduction

Myofascial pain is a common clinical entity with a high prevalence, ranging from 30% to 85% [1,2]. Myofascial pain may be involved in a variety of pain syndromes [3], including tension headaches [4], lower back syndromes [5], neck pain [6], and pelvic pain [7]. However, a definitive diagnosis and cause of myofascial pain is difficult to elucidate [6].

Several small studies and case reports elucidated the pathophysiology of myofascial pain beyond a purely muscle origin to include other soft tissue structures of the myofascial unit with its discrete layers: superficial fascia, deep fascia and muscle (epimysium, perimysium, endomysium) [8,9,10,11]. Prior attempts to characterize pain from specific fascial layers have shown that deep fascia may be associated with *aching* or *burning* pain, superficial fascia with *sharp or stiff* pain, and muscle as *dull* pain [12].

Ultrasound elastography has been used to demonstrate an increased stiffness and change in structure in the muscle of trigger points [13,14,15]. Changes in the structure and associated stiffness may relate to an increase in glycosaminoglycan content in the muscle near trigger points [16].

Pain syndromes may be related to increased thickness of the deep fascia [17] corresponding to an increase and change in extracellular matrix [18,19]. This aggregation of extracellular matrix in the deep fascia is termed densification [20]. The superficial fascia has a high density of innervation [21] and may be associated with myofascial pain [11]. In addition, centralized pain may occur alongside peripheral sensitization, especially in individuals with long-standing myofascial pain [22].

Understanding of how contributions from specific fascial layers of the myofascial unit (superficial fascia, deep fascia, and muscle) as well as how centralized processes correlate with the clinical presentation and pain characteristics of myofascial pain is still lacking. The objectives of this study were to delineate the contribution of specific fascial layers of the myofascial unit to myofascial pain and introduce the use of fascial layer-specific hydromanipulation (FLuSH) as a novel technique in the diagnosis and treatment of specific fascial layers contributing to myofascial pain.

## 2. Materials and Methods

This retrospective case series involved 20 consecutive adult patients (≥18 years) who underwent myofascial injections for the treatment of myofascial pain over a period of 5 consecutive months at an outpatient Physical Medicine and Rehabilitation practice. All analyzed data were assessed as part of the clinical routine during the treatment of myofascial pain using the FLuSH technique. Indications for injections included diagnosis of myofascial pain by a board-certified Physical Medicine and Rehabilitation physician, patient consent and desire to undergo injection treatment. The study was approved by IRB (Study 2019-92-CAS IRB) on 30/7/20. No patients were excluded from the analysis; all were provided with general informed consent per IRB protocol.

### 2.1. Fascial Layer Specific Hydromanipulation (FLuSH)

The myofascial treatment points were selected after a specific assessment process—the Fascial Manipulation^®^ method [8]—involving clinical examination of specific movements and palpatory verifications of specific myofascial points called Centers of Coordination (CCs) and Centers of Fusion (CFs). A CC corresponded to the convergence of the vectorial muscular forces acting on a joint in the deep fascia. A CF corresponded to the convergence of vectorial forces of planes located in the intermuscular septa, retinaculum, and ligaments. Palpation evaluation of these points included patient pain rate, radiation and the presence of tissue stiffness (corresponding to densifications) [23]. Dysfunctional segments were identified based on palpation and a hypothesis-driven differential by clinical history. Ultrasound elastography was used to verify stiffness [24,25].

The FLuSH technique was used to treat specific myofascial points with any of the following clinical features: a taut band, hypersensitivity, referred pain, stiffness, reproduction of pain [23,26]. In each session, an average of 3–5 myofascial points corresponding to CCs and CFs were injected.

After palpatory verification, the pain pressure threshold [27] was measured initially and after each injection at the site of injection using an analog algometer (Wagner instruments, model FDN 100, pressure surface 1 cm^2^ sensitivity +/1 1 N) [28]. The algometry reading was obtained by applying a perpendicular force to the skin with an increasing pressure of 1 N/s until the patient indicated that the feeling of pressure changed into a feeling of pain. The pressure at that moment was determined as the pain pressure threshold (expressed in N). Additionally, patients were asked to quantify the pain from 1–10 using the Visual Analog Scale (VAS) and characterize the pain by quality and radiation.

**Figure 1:**
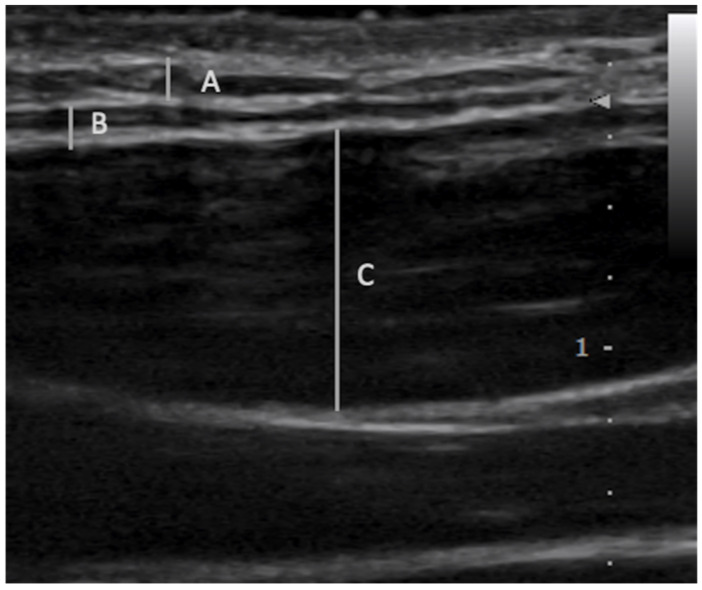
Fascial layer-specific hydromanipulation into specific fascial layers A: Superficial fascia B: Deep fascia C: Muscle.

The FLuSH technique involved using ultrasound guidance (Sonimage^®^ HS-1, Konica Minolta Corporation, Tokyo, Japan and a L18-4 transducer) [29] to inject approximately 0.5 mL of normal saline [30,31] into a specific fascial layer of the myofascial unit (Figure 1). The term “hydromanipulation” was used to differentiate this technique from “hydrodissection”, a technique used to treat nerve entrapments [32]. The needle was introduced in-plane to the transducer using aseptic technique. The needle was advanced towards the target fascial layer with the needle bevel positioned up, and the pressure of the injectate was used to open the deep fascia from the superficial fascia or the underlying muscle [33]. Similar to hydrodissection, the saline injectate, not the needle, was the tool used to manipulate the soft tissues. The needle was advanced slowly and visualized at all times during the procedure, particularly during needle advancement close to the target fascial layer, during which the physician continually injected fluid.

In the deep fascia, the same process was repeated with the needle targeting the hypoechoic extracellular matrix layers between organized fascia layers. The injectate was used to incrementally separate the soft tissues in front of the needle, followed by movement of the needle tip into the resultant fluid space. In the muscle and superficial fascia, a similar process was used to disperse saline to dilute aggregates in areas of stiffness corresponding to changes that were seen under elastography.

Deep fascia was injected first for pain characterized as *aching* or *burning*. Superficial fascia was injected first for pain described as *sharp or stiff*. Muscle was injected first for pain described as *dull* [12]. Each subsequent layer of fascia was injected sequentially (deep fascia, superficial fascia, muscle) until pain resolved or all layers were injected, whichever occurred first. Between injection of each fascial layer, pressure algometer was used to measure pressure at which the initial VAS score was reproduced. Meaningful change was considered greater than 5.8 N/cm^2^ of change based on statistically significant changes reported by prior studies on dry needling. Less than 5.8 N/cm^2^ of change was considered a non-response to injection of the specific fascial layer.

### 2.2. Data Collection

Clinical data were collected by chart abstraction. Specifically, pain descriptors, BMI, gender, pain pressure threshold, anatomic location/Fascial Manipulation^®^ points/planes were recorded.

### 2.3. Outcomes

Our primary endpoint was an increase in the pain pressure threshold as measured by pressure algometry. Any change in the pressure algometer greater than 5.8 N/cm^2^ of pressure in pressure tolerance was categorized as an improvement in pain response [28,34]. This composite outcome was chosen since the aim was to show the contribution (or lack thereof) of each fascial layer to myofascial pain.

### 2.4. Statistical Analysis

Discrete variables are expressed as frequency (percentage) and continuous variables as means with standard deviations.

## 3. Results

Table 1 provides an overview of the results. A total of 20 patients who underwent myofascial injections were included in this analysis (median age 50 ± 17 years; 80% females). A total of 87 points were injected using the FLuSH technique.

**Figure 2:**
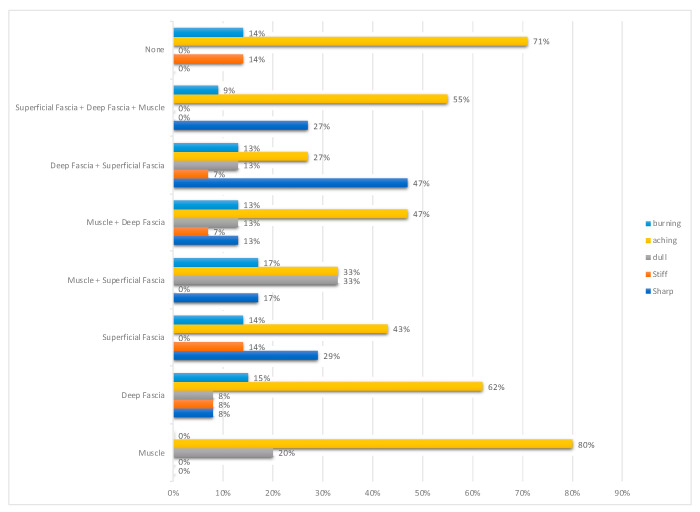
Descriptors of pain by fascial layers.

Pain descriptors are presented in Table 2 and Figure 2. The most common pain descriptor was aching. In a given patient, different combinations of fascial layers contributed to pain in treated myofascial points with an average of 3.0 ± 1.2 different combinations per patient. The most common combinations of fascial layer involvement were deep fascia alone in 23%, deep fascia and superficial fascia in 22%, and deep fascia and muscle in 18% of injected points. In varying combinations with other fascial layers, deep fascia was involved in 73%, muscle in 43%, and superficial fascia in 55% of all injected points.

Superficial fascia alone accounted for 14% of injected points. In 10% of injected points, pain pressure threshold did not change after treatment of all three layers. Overall, 75% of these non-responding points were in the upper trapezius area. Anatomic regions treated are listed in Table 3.

## 4. Discussion

Altered structure of the myofascial unit and the laydown of extracellular content in connective tissue is associated with pathology [17,35,36]. Fascia is an innervated and metabolically active tissue. The fascial system is highly abundant in nociceptive free nerve endings [37]. Entrapment or disruption of these nerve endings through excessive extracellular matrix laydown may lead to pain and dysfunction.

We postulate that similar to other modalities [17], the FLuSH technique uses the pressure of injected saline to mechanically disrupt the viscous nature of excess extracellular matrix content [38,39]. In addition, the FLuSH technique may serve to dilute the excess extracellular matrix content and various inflammatory mediators (catecholamines, neurogenic peptides, and cytokines) that lead to peripheral sensitization of the nociceptors [40] in myofascial pain. Clinically, adequate treatment of a specific fascial layer is seen as an increase in the pain pressure threshold post-injection and can serve as a diagnostic indicator for involvement of specific fascial layers. This specific diagnosis can guide further interventions like manual therapy after injection to target the specific fascial layers involved.

This study highlights and demonstrates an important phenomenon—myofascial pain may need to be treated as discrete entities—in a given person, myofascial points throughout the body may have pain generated by different combinations of layers of fascia. On average, patients had 3.0 different combinations of fascial layers contributing to myofascial pain. Given the multiple combinations of fascial layers involved in myofascial pain in a given person, it may be important to address each myofascial point discretely rather than as uniform entities in a given individual.

This study highlights the importance of superficial fascia. Superficial fascia was involved in myofascial pain in 55% of injected points, which is greater than that of muscle’s contribution (43%). This is no surprise as skin and superficial fascia have highly abundant nociceptive free nerve endings [37] and are the most highly innervated soft tissue [41]. Moreover, the superficial fascia acts as a conduit for nerves and is contiguous with the epimysium of muscle tissue [42]. Alteration of this paraneural tissue is seen in pathologies such as carpal tunnel syndrome [42]. This study further illustrates that the superficial fascia plays a large role in myofascial pain and warrants further examination.

In recent years, the deep fascia has been highlighted as a crucial contributor to myofascial pain [17,35,43], a fact further reinforced by this study. Deep fascia contributed to pain in 73% of injected points, the largest contributor in the myofascial unit. The deep fascia is also highly innervated [37]. There is evidence of alteration of c-fiber content with inflammation in the deep fascia [35]. Thickening of the deep fascia and the associated laydown of extracellular content is associated with myofascial pain [17,35,43]. Treatment using modalities and soft tissue manipulation has demonstrated reversal of deep fascial densification with resolution of pain [17]. The large contribution of deep fascia to myofascial pain may help elucidate the effectiveness of manual therapy (particularly when applied in a manner that addresses fascial planes) [29,44] in the treatment of myofascial pain.

The muscle has long been recognized as a source of pain in myofascial pain [3]. According to our results, muscle is indeed a partial contributor to pain in 43% of treated points. The mechanism has long been debated. Inflammatory mediators, catecholamines, neurogenic peptides, and cytokines may lead to sensitization of the nociceptor terminals in myofascial pain [40]. Excess extracellular matrix with aggregation of glycosaminoglycans has also been demonstrated in myofascial pain [16]. These aggregates are less likely to be associated with myofibrils [45,46] and more likely to occur in relation to connective tissue that accounts for 30% of muscle force [47]. Dry needling [15] may serve to disrupt the aggregates in these areas. We postulate that the FLuSH approach may serve to further disrupt the viscous nature of the glycosaminoglycan aggregates through the hydromechanical pressure of injected saline; the saline itself further serves to dilute the inflammatory chemicals and glycosaminoglycans present in muscle of myofascial pain points.

This study also highlights that both peripheral and centralized pain processes can co-exist in a given patient. Myofascial pain may occur from sensitization of the peripheral nociceptors as well as central nervous system sensitization [22,48,49]. We postulate that the 10% of treated points that did not respond to treatment of all three fascial layers of the myofascial unit likely represented a centralized pain process with a non-response of the pain pressure threshold (a phenomenon also seen in animal studies) [49]. Of these non-responding myofascial points, 75% occurred in the upper trapezius region, representing 38% of the upper trapezius’ treated myofascial points. These areas were subsequently and effectively treated using continuous touch [50].

Interestingly, most patients used the same pain descriptor across multiple treated points (Figure 2) with varying response in pain VAS score and presence of radiation (Table 1). Variations of pain descriptors based on fascial layer involvement were not consistent with data reported by Schilder et al. [12]. The discrepancy may be due to the multiple fascial layer involvement in myofascial pain as opposed to the individual, discrete layers studied in the Schilder et al. study. As low pain pressure threshold, pain quality, and non-radiation were not strong predictors of tissue layer origin, these parameters per se seem to not be useful for the decision regarding tissue layer treatment; however, further investigations need to be conducted.

Interestingly, FLuSH treatment of a few myofascial points increased sensitivity to pressure algometry after injection of a discrete layer but subsequently resolved with treatment of other affected fascial layers in the same point. A possible mechanism may be the amplification of pathologic mechanical disruptions of pathologic fascial layers when non-affected layers were treated, a process that possibly occurred through distention of associated skin ligaments [51] and other fascial continuities [42]. Another possible mechanism is the alteration of fascial structures by the needle itself [52].

This early report has limitations. Because of the retrospective single center study design, some of the most valuable ultrasound data were not routinely collected. We did not perform multivariable analysis because, due to the small number of patients and outcomes, the main purpose was not to derive a multivariable prediction model, but rather to provide an overview of the complex nature of myofascial pain. However, we strongly encourage such an effort using data of multiple centers, which would be much more generalizable. In addition, the low sample size limited the statistical evaluation and risk for type II error. Lastly, the study population only included patients within one region at one clinic without intermediate or long-term follow-up. Our cohort was highly selected and only included patients seeking specialized fascial treatment, whereas previous reports also included patients receiving standard trigger point injections. It would thus be interesting to validate our findings in a larger patient group within other healthcare settings. Furthermore, parameters of pain quality and duration of the response to specialized layer-specific myofascial injections using the FLuSH technique will have to be further investigated. Nevertheless, despite the preliminary nature of our data, this study highlights important characteristics of myofascial pain with insights into its complex nature.

## 5. Conclusions

This analysis provides insight into myofascial pain, including its characteristics, the contribution of fascial layers of the myofascial unit, and its complex nature. Within an individual, myofascial pain may originate from multiple fascial layers with different combinations of layers unique to each myofascial region and include centralized processes. Deep fascia played the largest role in contribution to pain. Superficial fascia and deep fascia in combination with other fascial layers also contributed significantly to myofascial pain. The ultrasound-guided FLuSH technique in the treatment of myofascial pain can be used to precisely and effectively treat myofascial pain and serve diagnostically for further treatment interventions including the treatment of centralized pain. Our findings, however preliminary, serve as a basis for further investigations concerning fascial layer-specific treatment of myofascial pain.

## Figures and Tables

**Table 1:** Number of discrete combinations of fascial layers contributing to pain in treated myofascial points across patients.

	Number of Treated Myofascial Points (% of All Treated Myofascial Points)
Muscle only	5 (6%)
Deep fascia only	18 (23%)
Superficial fascia only	11 (14%)
Muscle + Superficial Fascia	7 (9%)
Muscle + Deep Fascia	14 (18%)
Deep Fascia + Superficial Fascia	17 (22%)
Muscle + Deep Fascia + Superficial Fascia	7 (9%)
None	8 (10%)

**Table 2:** Number of discrete combinations of fascial layers contributing to pain across patients.

	Muscle Only	Deep Fascia Only	Superficial Fascia Only	Muscle + Superficial Fascia	Muscle + Deep Fascia	Deep Fascia + Superficial Fascia	Muscle + Deep Fascia + Superficial Fascia	None
VAS (mean ± standard deviation)	5.8 ± 1.8	6.1 ± 2.7	4.5 ± 1.7	6.0 ± 1.5	5.9 ± 1.8	5.8 ± 1.6	7.0 ± 1.7	5.9 ± 2.3
Radiating (% of layer specific radiation)	60%	89%	82%	100%	93%	73%	100%	0%

**Table 3:** Anatomical regions treated.

	Number of Treated Myofascial Points (% of All Treated Myofascial Points)
Trapezius	16 (18%)
Torso	14 (16%)
Arm	22 (25%)
Leg	35 (40%)
